# Pediatric procedural sedation and analgesia in the emergency department: surveying the current European practice

**DOI:** 10.1007/s00431-021-03930-6

**Published:** 2021-01-28

**Authors:** Cyril Sahyoun, Aymeric Cantais, Alain Gervaix, Silvia Bressan, Ruth Löllgen, Baruch Krauss, on behalf of the Pediatric Emergency Medicine Comfort and Analgesia Research in Europe (PemCARE) group of the Research in European Pediatric Emergency Medicine, Annick de Jaeger, Marianne Sjølin Frederiksen, Gérard Chéron, Katharina Röher, Florian Hoffmann, László Fodor, Idanna Sforzi, Itai Shavit, Zanda Pucuka, Vytenis Masilionis, Ruth Farrugia, Dorine Borensztajn, Ana Garrido, Diana Moldovan, Maria-Concepcion Miguez Navarro, Ioannis Orfanos, Anil Er, Murat Duman

**Affiliations:** 1 Division of Pediatric Emergency Medicine, Children’s Hospital of Geneva, Geneva University Hospitals, Rue Gabrielle-Perret-Gentil, 4, 1205 Geneva, Switzerland; 2 Department of Women’s and Children’s Health, University of Padova, Padova, Italy; 3 Pediatric Emergency Department, Inselspital, University Hospital, University of Bern, Bern, Switzerland; 4 Division of Emergency Medicine, Boston Children’s Hospital, Harvard Medical School, Boston, MA USA

**Keywords:** Pediatrics, Hypnotics and sedatives, Ambulatory surgical procedures, Procedural sedation and analgesia, Non-pharmacological approaches, Emergency medicine

## Abstract

Procedural sedation and analgesia outside the operating theater have become standard care in managing pain and anxiety in children undergoing diagnostic and therapeutic procedures. The objectives of this study are to describe the current pediatric procedural sedation and analgesia practice patterns in European emergency departments, to perform a needs assessment-like analysis, and to identify barriers to implementation. A survey study of European emergency departments treating children was conducted. Through a lead research coordinator identified through the Research in European Pediatric Emergency Medicine (REPEM) network for each of the participating countries, a 30-question questionnaire was sent, targeting senior physicians at each site. Descriptive statistics were performed. One hundred and seventy-one sites participated, treating approximately 5 million children/year and representing 19 countries, with a response rate of 89%. Of the procedural sedation and analgesia medications, midazolam (100%) and ketamine (91%) were available to most children, whereas propofol (67%), nitrous oxide (56%), intranasal fentanyl (47%), and chloral hydrate (42%) were less frequent. Children were sedated by general pediatricians in 82% of cases. Safety and monitoring guidelines were common (74%), but pre-procedural checklists (51%) and capnography (46%) less available. In 37% of the sites, the entire staff performing procedural sedation and analgesia were certified in pediatric advanced life support. Pediatric emergency medicine was a board-certified specialty in 3/19 countries. Physician (73%) and nursing (72%) shortages and lack of physical space (69%) were commonly reported as barriers to procedural sedation and analgesia. Nurse-directed triage protocols were in place in 52% of the sites, mostly for paracetamol (99%) and ibuprofen (91%). Tissue adhesive for laceration repair was available to 91% of children, while topical anesthetics for intravenous catheterization was available to 55%. Access to child life specialists (13%) and hypnosis (12%) was rare.

*Conclusion*: Procedural sedation and analgesia are prevalent in European emergency departments, but some sedation agents and topical anesthetics are not widely available. Guidelines are common but further safety nets, nurse-directed triage analgesia, and nonpharmacologic support to procedural sedation and analgesia are lacking. Barriers to implementation include availability of sedation agents, staff shortage, and lack of space.

**Table:** 

**What is Known:** • *Effective and prompt analgesia, anxiolysis, and sedation (PSA) outside the operating theatre have become standard in managing pain and anxiety in children undergoing painful or anxiogenic diagnostic and therapeutic procedures.* • *We searched PubMed up to September 15, 2020, without any date limits or language restrictions, using different combinations of the MeSH terms “pediatrics,” “hypnotics and sedatives,” “conscious sedation,” and “ambulatory surgical procedures” and the non-MeSH term “procedural sedation” and found no reports describing the current practice of pediatric PSA in Europe.*	
**What is New:** • *This study is, to the best of our knowledge, the first to shed light on the pediatric PSA practice in European EDs and uncovers important gaps in several domains, notably availability of sedation medications and topical anesthetics, safety aspects such as PSA provider training, availability of nonpharmacologic support to PSA, and high impact interventions such as nurse-directed triage analgesia.* • *Other identified barriers to PSA implementation include staff shortage, control of sedation medications by specialists outside the emergency department, and lack of space.*

**Supplementary Information:**

The online version contains supplementary material available at 10.1007/s00431-021-03930-6.

## Introduction

### Background

Managing pain, fear, and anxiety is a key factor in the well-being of children presenting for emergency care. These are often underrecognized and undertreated, with inadequately relieved pain and anxiety-producing physiological and psychological stress that have acute and long-term consequences [1–5]. Despite widespread efforts at enhancing pain and anxiety management, multiple barriers continue to exist between children and their comfort in medical settings.

Effective and prompt analgesia, anxiolysis, and sedation (collectively referred as PSA) outside the operating theater have become standard in managing pain and anxiety in children undergoing painful or anxiogenic diagnostic and therapeutic procedures. Many painful and anxiety-producing procedures do not require general anesthesia nor operating theater capabilities and can be safely, efficiently, and cost-effectively performed in an appropriate setting and at the patient bedside, such as in an emergency department (ED), an intensive care unit, a radiology, procedural sedation, gastroenterology, or hematology–oncology unit, with appropriately trained staff from multiple medical specialties. Such procedures include laceration repair, lumbar puncture, fracture reduction, abscess, pneumothorax and hemothorax drainage, thoracentesis, bone-marrow aspiration and biopsy, cross-sectional imaging requiring prolonged immobility, and gastrointestinal endoscopy, amongst others [6–10].

The North American experience with pediatric PSA is vast and widely reported in the scientific literature. Consortia also exist [11], where numerous hospitals pool their sedation data, allowing impactful research and guideline-generating studies to be conducted.

### Importance

Despite the fact that PSA is widely used in Europe and publications are increasing, the collective European PSA experience has not been thoroughly described. A 2008 survey of European pediatric EDs by Mintegui et al. looking at determinants of quality of care revealed that in 77% of the 54 surveyed pediatric EDs, sedation was provided by the pediatric ED staff, while 47% provided deep sedation, without further details on the subject [12]. The PIPER study group found that pediatric pain management is still sub-optimal in Italian EDs [13, 14]. National PSA guidelines for children have been developed in the UK, Italy, and the Netherlands [15–17].

### Goals of this investigation

The objectives of this study are to describe the current pediatric PSA practice patterns in European EDs, to perform a needs assessment-like analysis, and to identify barriers to the implementation of pediatric ED PSA. It is also anticipated that the study results would serve as an infrastructure for the creation of a European network of PSA experts and for the conduct of future prospective or interventional trials.

## Materials and methods

### Study design and setting

An online, multi-national, and cross-sectional survey of pediatric PSA practice in European EDs was undertaken between November 2019 and March 2020 (supplementary data). The study was endorsed by the Research in the European Pediatric Emergency Medicine (REPEM) network.

### Survey participants, content, development, distribution strategy, and target response rate

Through the mailing list of the REPEM network and through personal contacts of the REPEM network leadership, we identified a lead research coordinator for each of the participating countries from Europe and Israel. The UK and Ireland declined participation as a similar project targeting these two countries was being prepared at the time of our study.

The survey was drafted in English by the primary author. Similar to previously published survey studies, the questionnaire was repeatedly revised by the country lead research coordinators for language, grammar, content, comprehensiveness, and relevance, until consensus was reached about its applicability to the diversity of the region surveyed [18].

The survey started with a case vignette, then included questions spanning several domains:

1. Management of a theoretical patient requiring PSA
2. Medication availability and frequency of use
3. Characteristics of staff performing PSA and their training
4. Protocols and safety aspects
5. Nursing-directed triage protocols, topical anesthetics, and minor trauma care
6. Human resources around PSA
7. Barriers to implementation of PSA
8. Staff satisfaction with their site’s PSA efforts

Country leads were encouraged to reach the maximum number of sites possible, based on their knowledge of where children seek emergency care in their country, with the expectation that countries with a larger population would contribute proportionately more sites than countries with a smaller population. This outreach strategy was subsequently refined using a quota sampling method [19], whereby for countries with more than 20 million inhabitants (i.e., Italy, France, Germany, and Spain), the participation of 10 EDs was targeted. For countries with less than 20 million inhabitants, the participation of 5 EDs was targeted, unless the number of eligible EDs was less than 5 (e.g., 1 ED in Latvia, 2 in Malta), leading to a target denominator of 108 (Table 1) [20]. For calculation of the target response rate, the number of EDs exceeding the targeted number of responses per country was not considered.

**Table 1:** Characteristics of responding countries and sites

Country	Number of responses	Targeted number of responses	Target response rate	Mean number of children seen per site, in 2019 (95% CI)	Official board certification in PEM
Austria	5	5	100%	8110 (2116–14,104)	No
Belgium	5	5	100%	13,390 (4985–21,795)	No
Denmark	2	5	40%	5882 (1700–10,063)	No
France	11	10	100%	53,182 (40,118–66,246)	No
Germany	44	10	100%	10,477 (7344–13,611)	No
Greece	1	0	–	10,000	No
Hungary	3	5	60%	16,000 (2182–29,818)	No
Israel	9	5	100%	24,911 (15,644–34,178)	Yes
Italy	18	10	100%	27,931 (17,341–38,522)	No
Latvia	1	1	100%	63,000	No
Lithuania	3	5	60%	17,167 (1704–32,630)	No
Malta	1	2	50%	22,000	No
Netherlands	6	5	100%	4667 (1891–7443)	No
Portugal	10	5	100%	36,871 (25,623–48,119)	No
Romania	4	5	80%	21,978 (8746–35,210)	No
Spain	22	10	100%	37,294 (26,990–47,597)	No
Sweden	3	5	60%	32,000 (4270–59,730)	No
Switzerland	9	5	100%	28,087 (20,964–35,211)	Yes
Turkey	14	10	100%	88,284 (65,184–111,385)	Yes
Total	171	108	89%^a^	29,103 (24,647–33,559)	3/19

For countries with more than 20 million inhabitants (i.e., Italy, France, Germany, and Spain), the participation of 10 EDs was targeted. For countries with less than 20 million inhabitants, the participation of 5 EDs was targeted, unless the number of eligible EDs was less than 5 (e.g., 1 ED in Latvia, 2 in Malta), leading to an overall target denominator of 108. The number of EDs exceeding the targeted number of responses per country was not considered, in the calculation of the target response rate

^a^The overall response rate does not include the response from Greece as no country lead research coordinator was identified

Country leads were to decide on the strategy to approach sites (emails, phone calls, etc.), The survey was aimed at the clinical chief or person most aware of PSA efforts in each of the targeted sites, who was contacted by email or by phone and sent a weblink to the survey. One response was recorded per site. Through oversight by the primary author over a 3-month period and when necessary, country leads reviewed their national data with the purpose of reconciling potential input errors (identify duplicate entries, verify possibly mistyped information) and, if needed, contacted the survey participants. The survey was closed when every country lead, upon reviewing their country’s responses, believed that the number and type of responding sites were representative, or if they believed they had exhausted their ability to reach additional sites. Representation was defined as an adequate sample of the country’s main hospitals caring for children and was left to the discretion of each country lead coordinator.

We hypothesized that current PSA efforts are not uniform in European pediatric emergency care sites and that sites with different characteristics (number of pediatric patient treated per year, pediatric emergency medicine (PEM) as a recognized specialty) would be differently equipped and prepared for PSA (access to sedation medications, presence of protocols, and safety aspects).

### Statistical analysis

Study data were collected and managed using REDCap (Research Electronic Data Capture) electronic data capture tools hosted at the Geneva University Hospitals. REDCap is a secure, web-based software platform designed to support data capture for research studies providing (1) an intuitive interface for validated data capture, (2) audit trails for tracking data manipulation and export procedures, (3) automated export procedures for seamless data downloads to common statistical packages, and (4) procedures for data integration and interoperability with external sources [21].

Frequencies and percentages were used for categorical variables. Chi-square analysis, Fisher exact test, and non-parametric Kruskal–Wallis tests were used to identify statistically significant correlations, and *p* values were adjusted for multiple comparisons using the Benjamini–Hochberg method (using Stata Statistical Software: Release 14, College Station, TX: StataCorp LP). Given an expected large disparity in number of sites/country and of patients/site, and given that using the number of sites as the denominator would skew the data (some countries have the same total number of children represented but divided over a much larger number of sites), we chose to report the results as a proportion of the total number of children seen per year for domains involving patient-centered data (e.g., management of a theoretical patient, access to medications, characteristics of staff performing PSA, presented as percentages only). For domains involving site-centered data (frequency of use of sedation agents, existence of protocols), we report the results as a proportion of the total number of sites. For easier understanding of the data, we present percentages rounded to the nearest integer.

Completion of all items of the survey was required, and incomplete surveys were excluded from analysis.

### Ethics

Ethics Committee approval was granted by the Swiss Association of Ethics Committees (2018-01889). Consent was implied by participation.

## Results

### Respondents

**Fig. 1:**
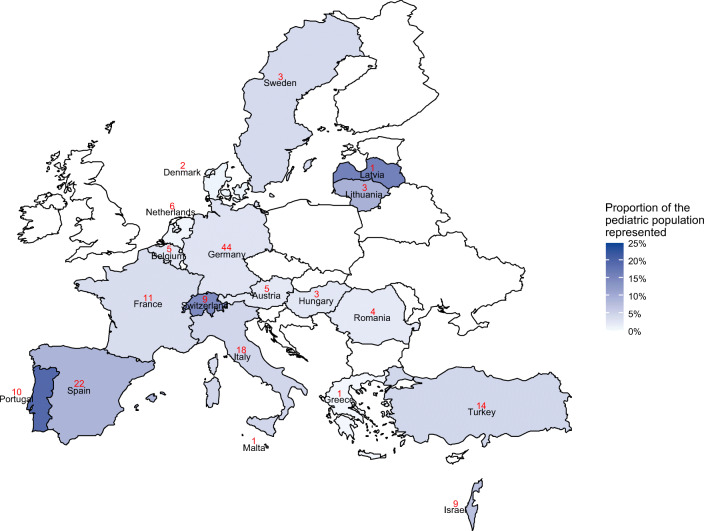
Geographic representation of survey participants and the proportion of the pediatric population represented (color gradient) by country, using the United Nations number of children 0–19 living in the country in 2019 as the denominator [72]

One hundred and seventy-one sites participated, representing 19 countries, with an overall target response rate of 89% (97/108), with Denmark (2/5, 40%), Malta (1/2, 50%), Hungary, Lithuania, and Sweden (each 3/5, 60%) the least represented (Fig. 1). The median number of participating sites per country was 5 (IQR 3–11). The mean number of children seen per year, per site, was 29,103 (95th CI 24,647–33,559). Ninety-one percent of the surveyed sites (156/171) took care of trauma patients. Eighty-three percent (142/171) of the sites were University Hospitals and/or Tertiary Care Centers (as locally defined). In 2019, the total number of children admitted to these sites was 4,976,581 (Table 1).

### Survey responses

1. *Management of a theoretical patient requiring PSA (as a proportion of children)*

A 4-year-old patient with a displaced forearm fracture requiring closed reduction and casting would be treated as follows: intravenous (IV) sedation in the ED in 61%, with nitrous oxide with or without a hematoma block and with or without intranasal (IN) fentanyl in 25%, and under general anesthesia in 25%. Children are treated without inhaled, IV, or IN medications in 8% of the cases (Table 2).

**Table 2:** Most commonly used methods for the management of a hypothetical patient presenting to a European emergency department with a forearm fracture requiring painful reduction and casting

Method	Number of sites (*n* = 156)^a^	Number of children represented (*n* = 4,578,308)
Intravenous sedation in the emergency department	84 (54%)	2,811,926 (61%)
Nitrous oxide +/− hematoma block +/− intranasal fentanyl)	33 (21%)	1,151,515 (25%)
Procedure done under general anesthesia by anesthesiologist	59 (38%)	1,120,033 (25%)
Intranasal fentanyl +/− intranasal midazolam	23 (15%)	458,096 (10%)
No inhaled, intravenous, or intranasal medications	8 (5%)	350,963 (8%)
Analgesia and transfer to a referral center	9 (6%)	158,000 (3%)
Intramuscular sedation in the emergency department	3 (2%)	45,200 (1%)

Ranked from most to least common. ^a^Fifteen sites representing 398,273 patients were excluded as they reported that they did not see trauma cases at their site (patients were referred elsewhere from scene of injury). The total is > 100% as several management options could be selected by a single site

## Sedation medication availability (as a proportion of children) and frequency of use (as a proportion of sites)

The following medications were available for use in children: midazolam (100%), ketamine (91%), and propofol (67%). Intranasal medications available for use in children included fentanyl (47%) and dexmedetomidine (10%). Nitrous oxide was available in 56% of sites and chloral hydrate in 42% (Table 3).

**Table 3:** Availability of selected medications and routes in European emergency departments

	As a proportion of sites surveyed	As a proportion of children represented
Systemic medications		
Ketamine		
- IV - IN - At least one route	152 (89%) 65 (38%) 154 (90%)	4,391,813 (88%) 1,358,347 (27%) 4,509,795 (91%)
Midazolam		
- IV - IN - PO - At least one route	161 (94%) 130 (76%) 110 (64%) 170 (99%)	4,718,081 (95%) 3,468,247 (70%) 2,731,395 (55%) 4,975,081 (100%)
Nitrous oxide - Excluding Turkey	93 (54%) 93/157 (59%)	2,770,386 (56%) 2,770,386/3,740,599 (74%)
Propofol IV	123 (72%)	3,319,582 (67%)
Fentanyl		
- IV - IN	133 (78%) 100 (58%)	3,788,481 (76%) 2,355,686 (47%)
Etomidate IV	60 (35%)	1,554,819 (31%)
Dexmedetomidine IN	18 (10%)	476,089 (10%)
Chloral hydrate		
- PO - PR - At least one route	54 (32%) 46 (27%) 74 (43%)	1,472,314 (30%) 1,311,395 (26%) 2,086,532 (42%)
Topical anesthetics and tissue adhesive		
Topical anesthetics		
- For laceration carea - For intravenous catheterization	109 (68%) 110 (64%)	3,313,787 (71%) 2,756,071 (55%)
Tissue adhesive^b^	147 (91%)	4,209,719 (91%)

*IV* intravenous, *IN* intranasal, *PO* per Os, *PR* per rectum

^a^Denominators are 160 sites and 4,688,808 children as 11 sites stated they did not care for lacerations for this question

^b^Denominators are 161 sites and 4,641,808 children as 10 sites stated they did not care for lacerations for this question

Where available, intravenous sedation was used twice a week or less in 53% (77/146) of the sites and 1 to 2 times a day or more in 20% (30/146). Nitrous oxide was used twice a week or less in 33% (29/88) and 1 to 2 times a day or more in 39% (34/88). Equimolar 50% nitrous oxide/50% oxygen was the most widely used mixture, with 11% (10/88) of the sites sanctioned to use 70%/30%.

## Characteristics of staff performing PSA (as a proportion of children) and training (as a proportion of sites)

Children were sedated by general pediatricians in 82% of cases, followed by pediatric emergency physicians in 70%, anesthesiologists in 36%, and pediatric intensivists in 29%.

Specific PSA courses, in addition to pediatric advanced life support (ALS) courses, were required for the staff administering PSA in 48% (82/171) of the sites, while a specific number of supervised PSA was required in 43% (73/171) before performing independently. In 37% (63/171) of the sites, the entire physician staff performing PSA were pediatric ALS course certified, and in 16% (28/171), less than a quarter were certified.

Trainees were allowed to administer PSA in 62% (107/171) of the sites, of which 76% (81/107) only if in their senior year and 24% (26/107) at any time during their training.

Emergency medicine was a board certification in 84% (16/19) and pediatric emergency medicine in 16% (3/19) of the countries surveyed (Table 1).

## Protocols and safety aspects (as a proportion of sites)

General safety and monitoring guidelines (detailing indications and contraindications for sedation, staff required to be present in the room, monitoring requirements) were present in 74% (127/171) and pre-procedural checklists (a specific checklist of material, adjunct medications, and information that needed to be prepared or obtained in preparation for the sedation) in 51% (87/171). Capnography (via nasal–oral cannula) was available in 46% (79/171) of the sites. During PSA with IV ketamine, physicians administered the medication in 59% (89/152), nurses in 33% (50/152), and either physician or nurse in 9% (13/152).

## Nurse-directed triage analgesia protocols (as a proportion of sites), topical anesthetics, and minor trauma care (as a proportion of children)

Nurse-directed triage analgesia protocols (a protocol or standing order allowing nurses to give analgesics at triage without prior medical prescription) were in place in 53% (90/171) of sites. Of those, the protocol included paracetamol in 99% (89/90), ibuprofen or similar non-steroidal anti-inflammatory drug in 91% (82/90), an oral opiate in 22% (20/90), and IN fentanyl in 12% (11/90). Topical anesthesia for lacerations (lidocaine, epinephrine/adrenaline, tetracaine, or similar) was available to 71% of the children and for IV catheterization (Eutectic Mixture of Local Anesthetics, EMLA or similar) to 55%. Tissue adhesive for laceration repair (tissue adhesive such as Dermabond, SurgiSeal) was available to 91% of the children.

## Human resources around PSA (as a proportion of sites)

The availability, at any time of the day, of a physician able to perform PSA was 34% (58/171) for single coverage (one individual present at any given time) and also 34% (58/171) for double coverage (two individuals present at any given time). Nurse availability was 28% (48/171) for single coverage and 34% (59/171) for double coverage. ED physicians sedated outside the ED in 32% (55/171) of the sites. A formal medical sedation service (a team sedating patients from different services of the hospital, such as ward, radiology, or other interventional services) was available in 52% (89/171), staffed by anesthesiologists in 79% (70/89), pediatric intensive care medicine physicians in 41% (37/89), pediatric emergency medicine physicians in 13% (12/89), and general pediatricians in 12% (11/89) of the sites.

Child life specialists (CLS) and hypnosis were available to 13 and 12% of the children, respectively. The Netherlands had the most availability of CLS (67%, 4/6) and Belgium the greatest availability of hypnosis (80%, 4/5).

## Barriers to implementation of PSA (as a proportion of sites)

Physician and nursing staff shortages were reported in 73% (125/171) and 72% (123/171) of sites and lack of physical space in 69% (118/171) of sites as barriers to PSA. Anesthesiologists controlled or restricted ketamine and propofol use (defined as the ED not being free to create a protocol and use the medication without direct supervision or official approval) in 25% (42/171) and 44% (75/171) of the sites, respectively. Eighty-five percent (146/171) of respondents agreed that ketamine was a useful agent for PSA in the ED.

## Staff satisfaction around PSA (as a proportion of sites)

Sixty-five percent (111/171) of respondents stated being satisfied with their site’s management of pain and anxiety of children during procedures. Of respondents who answered the question, main reasons for dissatisfaction included lack of training and of protocols in 68% (19/28), staff unavailability in 18% (5/28), and medication unavailability in 14% (4/28).

## Other notable findings (as a proportion of sites)

Relationships between predictors and outcomes were analyzed. Sites in the highest tercile of patients visits per year were more likely to have nurse-directed triage protocols in place (70% vs. 47% in the middle tercile and 41% in the lowest tercile, adjusted *p* = 0.055). In addition, sites where IV sedation was most frequently performed had a higher likelihood of having several safety protocols in place (93% vs. 74 to 79%), but this was not statistically significant (adjusted *p* = 0.701). Having an official board certification in pediatric emergency medicine (PEM) correlated with a higher prevalence of specific PSA curricular (adjusted *p* = 0.049), requirement for supervised PSA (adjusted *p* = 0.007), and also correlated with the absence of a medical sedation service (adjusted *p* = 0.039) (Table 4).

**Table 4:** Relationships between European emergency department characteristics and existence of clinical components spanning safety, technology, and human resources around pediatric procedural sedation and analgesia

	Number of children seen per year (terciles)					Frequency of IV sedation						Belonging to a university or tertiary care center				Existence of a board certification in pediatric emergency medicine			
	50–12,000 *n* = 60	12,000–31,000 *n* = 55	31,000 to max *n* = 57	*p*	Adjusted *p*	Less than 1/week *n* = 43	About 1–2/week *n* = 34	About 2–6/week *n* = 39	1/day or more *n* = 30	*p*	Adjusted *p*	No *n* = 29	Yes *n* = 142	*p*	Adjusted *p*	No *n* = 139	Yes *n* = 32	*p*	Adjusted *p*
Specific PSA curriculum	22 (37%)	27 (49%)	33 (58%)	0.26	0.555	23 (54%)	16 (47%)	21 (54%)	17 (57%)	0.485^a^	0.701	13 (45%)	69 (49%)	0.445^a^	0.712	60 (43%)	22 (69%)	0.015^a^	0.049^a^
Specific number of supervised PSA	26 (44%)	22 (40%)	25 (44%)	0.687	0.687	18 (42%)	12 (35%)	22 (56%)	17 (57%)	0.426^a^	0.701	12 (41%)	61 (43%)	0.999^a^	0.999	51 (37%)	22 (69%)	0.001^a^	0.007^a^
General safety rules for administering sedation	43 (73%)	39 (71%)	45 (80%)	0.11^a^	0.392	33 (77%)	27 (79%)	29 (74%)	28 (93%)	0.361^a^	0.701	18 (62%)	109 (77%)	0.189^a^	0.712	98 (71%)	29 (91%)	0.058^a^	0.108
PSA checklist	27 (46%)	27 (49%)	33 (58%)	0.14^a^	0.392	25 (58%)	17 (50%)	22 (56%)	20 (67%)	0.848^a^	0.919	15 (52%)	72 (51%)	0.526	0.712	66 (48%)	21 (66%)	0.125^a^	0.203
Capnography	31 (53%)	22 (40%)	26 (46%)	0.404	0.589	17 (40%)	15 (44%)	24 (62%)	18 (60%)	0.132	0.510	11 (38%)	68 (48%)	0.327	0.712	59 (43%)	20 (63%)	0.04	0.104
Medical sedation service	30 (51%)	32 (58%)	27 (47%)	0.506	0.590	18 (42%)	23 (68%)	19 (49%)	14 (47%)	0.138	0.510	13 (49%)	76 (54%)	0.393	0.712	79 (57%)	10 (31%)	0.009	0.039^a^
Nurse-directed triage analgesia	24 (41%)	26 (47%)	40 (70%)	0.004	0.056	20 (47%)	18 (53%)	20 (51%)	19 (63%)	0.561	0.719	10 (35%)	80 (56%)	0.032	0.416	70 (50%)	20 (63%)	0.215	0.291
Hypnosis	12 (20%)	4 (7%)	9 (16%)	0.136	0.392	5 (12%)	6 (18%)	6 (15%)	7 (23%)	0.608	0.719	4 (14%)	21 (15%)	0.89	0.999	21 (15%)	4 (13%)	0.707	0.707
Child life therapists	5 (9%)	2 (4%)	10 (18%)	0.044	0.308	5 (12%)	3 (9%)	4 (10%)	4 (13%)	0.946	0.946	2 (7%)	15 (11%)	0.548	0.712	13 (9%)	4 (13%)	0.592	0.641

*p* value refers to chi-square test. Adjusted *p* value refers to the adjustment for multiple comparisons by the Benjamini–Hochberg method

*PSA* procedural sedation and analgesia, *IV* intravenous

^a^Fisher exact test

## Discussion

**Fig. 2:**
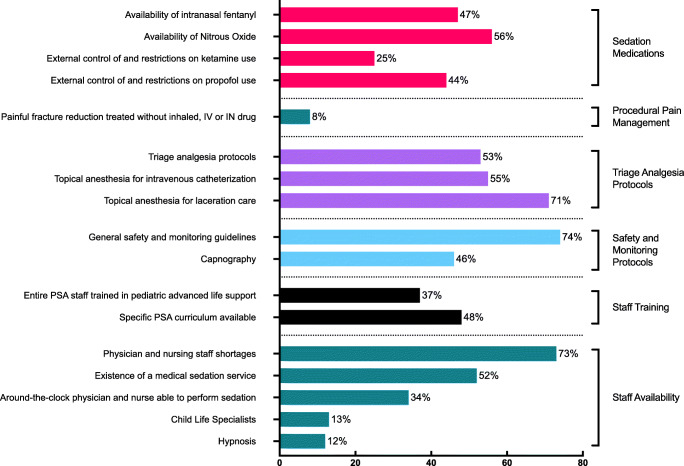
Incidence of identified gaps in selected domains. PSA, procedural sedation and analgesia; IV, intravenous; IN, intranasal

In this multi-national European survey, we investigated the current PSA practice available to children presenting for emergency care to hospitals across the region and found that although PSA is widely practiced in European EDs and general safety guidelines are common, some sedation medications, topical anesthetics, requirement for pre-procedural checklists and universal pediatric ALS training, nurse-directed triage analgesia protocols, and access to CLS and hypnosis are lacking. Barriers to PSA implementation also include staff shortage, control of sedation medications by specialists outside the emergency department, and lack of space (Fig. 2). A summary of all identified gaps and associated recommendations is provided in Table 5.

**Table 5:** Summary of existing gaps in pediatric procedural sedation and analgesia (PSA) practice in European emergency departments (italics represent the explanation of the recommendation in nontechnical terms)

1. Sedation medications • Gap: Restricted pharmacopeia with limited appropriate medication options, in part due to external constraints: i. Limited availability of intranasal fentanyl and nitrous oxide ii. Restrictions on use of Ketamine and Propofol • Recommendation: PSA sites should work on increasing the availability of the full range of PSA agents, prioritizing intranasal fentanyl, nitrous oxide and ketamine, in order to deliver optimal care for patients. • *Fentanyl is a medication used for immediate relief from severe pain. Its nasal spray form is safe and makes the use of needles unnecessary. Nitrous oxide is a widely available gas used to sedate anxious children for mild–moderately painful procedures. Ketamine is a safe and highly effective medication in emergency sedation, especially for very painful procedures. Increasing the availability of these medications and training for emergency department/site staff in their use is an essential part of improving the care of children in emergency situations.* 2. Procedural pain management • Gap: Lack of adequate pain control for children undergoing painful procedures • Recommendation: Every child should have an appropriate assessment of their baseline pain, an assessment of the anticipated pain and anxiety of the procedure, and a sedation plan for providing adequate relief of pain and anxiety. • *Children continue to receive inadequate treatment for painful procedures. All children should receive adequate control of their pain and anxiety during emergency department procedures. This requires both the availability of appropriate medications for sedation and analgesia and comprehensive staff training.* 3. Triage analgesia protocols • Gap: Limited availability of nurse-directed triage analgesia protocols and limited use of topical anesthetics • Recommendation: Universal establishment of triage analgesia protocols for systemic analgesics and for topical anesthetics for venipuncture, intravenous catheter placement, and laceration repair. • *The patient experience is improved by the use of protocols for the triage area, which allow nurses to rapidly and safely treat children’s pain using pain medications, as well as to prepare patients for needle sticks or wound repair, using anesthetic ointments or creams, without having to consult a physician. The use of a topical gel applied to the laceration before suturing allows many wounds to be stitched without discomfort or the need for an injection of lidocaine. The use of a topical cream before a needle stick for a blood draw or placement of an intravenous line also helps minimize the discomfort or pain experienced by the patient. We advocate for the universal use of these measures.* 4. Safety and monitoring protocols • Gap: Limited implementation of standardized PSA safety and monitoring guidelines • Recommendation: Universal implementation of evidence-based PSA guidelines (risk assessment and contraindications to PSA, fasting status, preparation for adverse events, continuous oxygenation and ventilation monitoring, post-procedural care, and discharge criteria). • *We encourage the universal use of PSA guidelines and continuous electronic patient safety monitoring which help ensure maximum safety during PSA through early recognition and management of the adverse effects related to treatment.* 5. Staff training • Gap: Limited staff training in pediatric advanced life support and in PSA skills • Recommendation: Physicians administering PSA should be trained in pediatric advanced life support. Specific PSA curricular training (such as didactics on pain and anxiety recognition, assessment, and management, evidence-based utilization of analgesics and sedatives, incorporation of simulation PSA training, and implementation of a rigorous, supervised sedation practice) should also be instituted to provide safe and effective PSA. • *All physicians performing sedation should be trained in rescuing patients from the adverse effects of sedation, should they occur. We advocate for universal training in pediatric life support courses as well as specific analgesia and sedation training to improve the patient experience.* 6. Staff availability • Gap: Limited availability of PSA-trained staff • Recommendation: Emergency sites should employ developmentally appropriate approaches to frightened children and devise a plan for 24-h access to sedation services. In resource-limited settings, this can be achieved using multispecialty partnerships. • *The management of pain, fear and anxiety in children should be consistent whether during normal daytime hours, on the weekend or during the night. In hospitals that have too low a volume to dedicate the care of such issues to one specialty, partnerships with other specialties should be sought to ensure around-the-clock adequate procedural pain relief and sedation care for children.*	

### Sedation medication availability

The ability to receive prompt, safe, and effective procedural pain relief is paramount to children in emergencies. Ketamine, a dissociative sedative that has consistently been shown to be safe and effective was available to 91% of the children represented by this study. Propofol and combinations of propofol and ketamine (“ketofol”) have also been shown to be safe and effective PSA agents [22–24]. In our study, propofol was available to two thirds of the children represented, and we did not inquire about the use of ketofol. Nitrous oxide (N_2_O), a safe, generally available, and useful agent in pediatric PSA, ubiquitously found in anesthesia machines in the operating theater, was surprisingly available to only half of the children. Excluding Turkey (where nitrous oxide is not available in emergency settings and which constituted a quarter of the children represented by the survey), the availability of N_2_O increases to three quarters. Further inquiry would be necessary to understand why a quarter of children do not have access to N_2_O, a question this survey did not address. Intranasal dexmedetomidine has been gaining adoption in the recent years [25–29], as an efficacious alternative to midazolam for non-painful procedures, with the added advantage, of not being painful during administration unlike midazolam [30], its effects mimicking sleep, being safe [31], and being protective in anesthetic neurotoxicity [32–34]. Its availability to only one tenth of the children represented by our study appears reflective of only a slow rise in popularity, perhaps secondary to a longer onset and duration of action than midazolam, which makes it less useful in environments where throughput is important, or secondary to physician familiarity and preference for older medications such as midazolam. Chloral hydrate, despite concerns over its safety and toxicity, was available to a third of children [35, 36].

### Safety aspects and training of staff performing PSA

A multitude of international regulatory entities and individual experts have disseminated safety guidelines around PSA, guiding practitioners on topics including staff training, safety, and the use of monitoring [15, 37–46]. In our study, we found that although general safety and monitoring guidelines were in place in three quarters of the sites, only a third had the entirety of the staff performing PSA certified in a pediatric ALS course. When excluding sites that allowed trainees to perform sedation, this proportion barely increases to 42%. This number is of concern and appears to be another “low hanging fruit” for improving safety of PSA and complying with the guidelines mentioned above. Indeed, practicing a skill outside of guidelines, particularly when at a low frequency and without the proper training, imposes a large amount of risk on the patient’s well-being.

### Nurse-directed triage protocols and topical anesthetics

Nurse-directed triage protocols have been shown to improve time to analgesia [47–51]. In our study, only half of the sites had access to such protocols. For the sites that do not have such protocols in place, this may represent one of the areas for greatest improvement in the provision of prompt and effective analgesia. Another area for improvement is the use of topical anesthesia for venipuncture and prior to laceration repair. Despite the fact that the literature has repeatedly shown their benefit [52–57], their availability in our cohort was limited. Even excluding Turkey, where the rate of topical anesthesia for wound care is 42% and 5% for venipuncture, the overall rates of our cohorts only increase to 70% and 71%, respectively (with the least access in Hungary, Malta, Portugal, and Romania).

### Staff shortage and lack of space

PSA is a resource-consuming task often requiring pre-procedure preparation and post-procedure monitoring. In addition to the shortages and lack of space reported above, physician availability was a matter of concern in three quarters of cases, and nursing availability in just above 50%. For sites moving to a new site or planning on building new EDs, including a dedicated procedural room or rooms in the architectural plans would provide an answer to the lack of space reported.

### Child life therapists and hypnosis

Nonpharmacologic support to PSA such as the utilization of CLS and hypnosis have also been shown to successfully reduce pain, stress, and anxiety in children undergoing procedures [58–68]. The American Academy of Pediatrics has also stated that the provision of CLS is a quality benchmark of an integrated patient and family-centered health care system, a recommended component of medical education, and an indicator of excellence in pediatric care [69]. In Europe, only a few countries appear to train and utilize CLS. In the UK, the National Association of Health Play Specialists aims to “promote the physical and mental well-being of children and young people who are patients in hospitals, hospice or receiving medical care at home” [70]. In the Netherlands, CLS are currently working on obtaining official professional status [71]. In our study, CLS and hypnosis were rarely available. Underlying reasons may include cost–benefit financial considerations and need for prioritization of resources, particularly in limited resource settings (hiring CLS vs. physicians or nurses able to perform IV sedation), inadequate knowledge about the added benefit of such approaches, cultural reasons (continued delegation of such tasks to medical and nursing staff), lack of available training and lastly, scarcity of published research in children. The reasons for the scarcity of CLS in Europe merit further investigation, in an effort to continue optimizing pediatric comfort and procedural care.

### Limitations

Our study has several limitations. Although a large number of European countries participated in this study, not all 47 countries are represented, despite multiple attempts to recruit country leads for each country. The reasons include the lack of representation of some of those countries in the REPEM network or other professional PEM specialty networks. This may have led to a biased representation of the current practice variation of PSA across Europe, favoring countries with representation in research platforms such as the REPEM network. The creation of a complete contact registry and recruitment of additional members from all European countries for the REPEM network will be useful for future collaboration and research.

The European PEM landscape is hardly uniform. Levels of specialty development and dedicated pediatric emergency care access differ widely. Although implementation of PEM as an official specialty has been enacted and dedicated PEM departments have been created in a small number of countries (e.g., Israel, Switzerland, Turkey), it has not in others and, accordingly, understanding of PEM-related terms and issues by respondents might have been inhomogeneous. Also, some countries do not belong to the European Union and legislation and access to resources such as medications might be heterogeneous. In addition, most sites (83%) in this study belong to a University Hospital or are tertiary care centers. As the proportion of children treated in such hospitals vs. small community hospitals may vary from country to country, our study may not be fully representative of the reality of children treated on an emergency basis and possibly overestimates the availability of resources.

The survey methodology itself is subject to several biases. Sampling bias, where the person targeted by the survey is possibly not the most appropriate to answer the survey, is one. However, the country lead research coordinator strategy detailed above was implemented to avoid such bias by targeting specific individuals in the field of PEM. Nonresponse bias (where subjects who do not respond to surveys differ significantly from those who do) was in part addressed by repeat reminders to complete the survey, but as above may lean the study results towards countries and EDs with more established programs in pediatric emergency medicine. In this respect, our findings may not be generalizable across different settings.

## Conclusion

Despite PSA being widely practiced in Europe, some sedation agents, topical anesthetics, access to CLS, and hypnosis are not widely available. PSA guidelines are common but pre-procedural checklists, universal pediatric ALS training, and nurse-directed triage analgesia protocols are limited. Identified barriers to PSA implementation include availability of sedation agents, staff shortage, control of sedation medications by specialists outside the emergency department, and lack of space. The results of this survey can be used as a needs assessment to bridge the gap towards best practice in European PSA and serve as a ground for collaboration, guideline creation, curriculum design, and future interventional trials amongst European EDs caring for children.

## Supplementary Information

ESM 1 (PDF 63.4 kb)

## Data Availability

Request for access to the data should be made to the corresponding author at cyril.sahyoun@hcuge.ch. Data could be made available provided the applicant has appropriate ethics approval and approval from the authors, and a data transfer agreement is created.

## Abbreviations

ALS: Advanced life support
CLS: Child life specialists
ED: Emergency department
IN: Intranasal
IV: Intravenous
PED: Pediatric emergency department
PSA: Procedural sedation, anxiolysis, and analgesia
