# Correction to: Pediatric procedural sedation and analgesia in the emergency department: surveying the current European practice

**DOI:** 10.1007/s00431-021-03980-w

**Published:** 2021-02-13

**Authors:** Cyril Sahyoun, Aymeric Cantais, Alain Gervaix, Silvia Bressan, Ruth Löllgen, Baruch Krauss, on behalf of the Pediatric Emergency Medicine Comfort and Analgesia Research in Europe (PemCARE) group of the Research in European Pediatric Emergency Medicine (REPEM) network, Annick de Jaeger, Marianne Sjølin Frederiksen, Gérard Chéron, Katharina Röher, Florian Hoffmann, László Fodor, Idanna Sforzi, Itai Shavit, Zanda Pucuka, Vytenis Masilionis, Ruth Farrugia, Dorine Borensztajn, Ana Garrido, Diana Moldovan, Maria-Concepcion Miguez Navarro, Ioannis Orfanos, Anil Er, Murat Duman

**Affiliations:** 1 Division of Pediatric Emergency Medicine, Children’s Hospital of Geneva, Geneva University Hospitals, Rue Gabrielle-Perret-Gentil, 4, 1205 Geneva, Switzerland; 2 Department of Women’s and Children’s Health, University of Padova, Padova, Italy; 3 Pediatric Emergency Department, Inselspital, University Hospital, University of Bern, Bern, Switzerland; 4 Division of Emergency Medicine, Boston Children’s Hospital, Harvard Medical School, Boston, MA USA

**Correction to: European Journal of Pediatrics**

10.1007/s00431-021-03930-6

In the Acknowledgement section of the original published version of the above article, the complete list of the **Country Lead Research Coordinators of PemCARE group of the REPEM network** were not mentioned thus the Acknowledgments has been corrected as follows:

**Acknowledgments** The authors wish to thank each country lead research coordinator and each of the survey participants who gave their time to diffuse or complete the survey.

**Country Lead Research Coordinators of PemCARE group of the REPEM network**

Annick de Jaeger, Ghent University Hospital, Belgium

Marianne Sjølin Frederiksen, pediatric emergency department, Copenhagen University Hospital, Herlev, Denmark

Gérard Chéron, Necker Enfants Malades Hospital, Université de Paris, France

Katharina Röher, University Medical Center Hamburg-Eppendorf, Hamburg, Germany

Florian Hoffmann, Dr. von Hauner Children’s Hospital, Ludwig-Maximilians-University, Munich, Germany

László Fodor, Fejér County Szent György University Teaching Hospital, Székesfehérvár, Hungary

Idanna Sforzi, Anna Meyer Children's Hospital, Florence, Italy

Itai Shavit, Rambam Health Care Campus, Israel

Zanda Pucuka, BKUS Children Clinical University Hospital, Latvia

Vytenis Masilionis, The Hospital of Lithuanian University of Health Sciences, Klauno Klinikos, Kaunas, Lithuania

Ruth Farrugia, Mater Dei Hospital, Msida, Malta

Dorine Borensztajn, Erasmus MC-Sophia Children's Hospital, Rotterdam, The Netherlands.

Ana Garrido Centro Hospitalar Tamega e Sousa, Portugal

Diana Moldovan, Emergency Department, Tirgu Mures Emergency Clinical County Hospital, Targu Mures, Romania

Maria-Concepcion Miguez Navarro, Hospital General Universitario Gregorio Marañón, Madrid, España

Ioannis Orfanos, Department of Pediatrics, Skane University Hospital, Lund University, Sweden

Anil Er, Dokuz Eylul University School of Medicine, Izmir, Turkey

Murat Duman, Dokuz Eylul University School of Medicine, Izmir, Turkey

The original article has been corrected.

