# Supplementary material for: Pediatric procedural sedation and analgesia in the emergency department: surveying the current European practice
Source: Eur J Pediatr. 2021 Jan 28;180(6):1799–813. doi: 10.1007/s00431-021-03930-6 (PMC8105204; doi:10.1007/s00431-021-03930-6)
Supplement: Supplementary file 1 — (PDF 63.4 kb) [file 431_2021_3930_MOESM1_ESM.pdf]

European PSA survey

Dear colleague,

Thank you for your time answering this survey on Pediatric Procedural Sedation and Analgesia (PSA) in the Emergency Department (ED).

Our aims are to feel the pulse of PSA in Europe, to create a network of sedation experts, and together to find opportunities for collaborative research, for training, and for enhancing safety in PSA.

Cyril Sahyoun, MD, Pediatric Emergency Medicine, Geneva University Hospitals

Disclaimer on identifiable information: The data will be securely stored and no data will be communicated outside of the scope of this survey. Once data analysis is complete, likely within the year, the data will be destroyed. Country and hospital name will be used to compare answers by country and to track double entries.

If you have any questions regarding the data, please contact me at cyril.sahyoun@hcuge.ch

---

Please note that unless noted, the questions below refer strictly to the care of children who are presenting to the Emergency (or Pediatric Emergency) Department.

Case:

A 4 year old girl arrives at your emergency department with a displaced fracture of the radius and ulna, without vascular or neurological deficits. The fracture requires closed reduction and casting (no surgical material needs to be placed), which will be very painful.

What are the way or ways this case is most commonly managed, in your department (multiple responses allowed)?

- ☐ Analgesia and transfer to a referral center
- ☐ Procedure done under general anesthesia by anesthesiologist
- ☐ IntraVENOUS sedation in the emergency department
- ☐ IntraMUSCULAR sedation in the emergency department
- ☐ Nitrous oxide +/- hematoma block +/- intranasal fentanyl
- ☐ IntraNASAL fentanyl +/- intraNASAL midazolam
- ☐ No inhaled, intravenous or intranasal medications
- ☐ Other
- ☐ We do not see Trauma cases at our hospital (transported elsewhere from site of injury)

How is this patient otherwise managed?

____

What molecule(s) and route(s) of administration are available, for the PURPOSE of Procedural Sedation and Analgesia on children, in your ED (multiple answers and routes possible) ?

	Inhaled	Oral	IV	IM	Intranasal	Intrarectal	Not available in our ED
Nitrous oxide (inhaled)	☐	☐	☐	☐	☐	☐	☐
Midazolam	☐	☐	☐	☐	☐	☐	☐
Fentanyl	☐	☐	☐	☐	☐	☐	☐
Morphine	☐	☐	☐	☐	☐	☐	☐
Ketamine	☐	☐	☐	☐	☐	☐	☐
Propofol	☐	☐	☐	☐	☐	☐	☐
Dexmedetomidine	☐	☐	☐	☐	☐	☐	☐
Chloral hydrate	☐	☐	☐	☐	☐	☐	☐
Etomidate	☐	☐	☐	☐	☐	☐	☐

Who physically administers the intraVENOUS medications for PSA, in your ED?

	Physician	Nurse	Either one	Not available in our ED
Midazolam	◯	◯	◯	◯
Ketamine	◯	◯	◯	◯
Propofol	◯	◯	◯	◯
Dexmedetomidine	◯	◯	◯	◯
Other intraVENOUS medications	◯	◯	◯	◯

What other intraVENOUS medication is that?

____

To the best of your knowledge or estimation, how many dissociative (KETAMINE) and deep sedations (PROPOFOL, KETOFOL, etc) are performed?

Please note, first set of answers is per WEEK, second is per DAY

- ◯ None, we do not use either molecule
- ◯ Less than 1/WEEK
- ◯ about 1-2/WEEK
- ◯ about 2-4/WEEK
- ◯ about 4-6/WEEK
- ◯ about 1-2/DAY
- ◯ about 2-4/DAY
- ◯ about 4-5/DAY
- ◯ > 5/DAY

To the best of your knowledge or estimation, how many NITROUS OXIDE sedations are performed?

Please note, first set of answers is per WEEK, second is per DAY

- ◯ None, we do not use nitrous oxide
- ◯ Less than 1/WEEK
- ◯ about 1-2/WEEK
- ◯ about 2-4/WEEK
- ◯ about 4-6/WEEK
- ◯ about 1-2/DAY
- ◯ about 2-4/DAY
- ◯ about 4-5/DAY
- ◯ > 5/DAY

What concentration Nitrous Oxide do you have available in the ED?

- ◯ 50/50 % nitrous/oxygen
- ◯ Up to 70/30 % nitrous/oxygen

Approximately, and on a typical weekday, during normal DAY hours, how many PHYSICIANS are usually present to perform INTRAVENOUS PSA? How about the number of nurses able to monitor INTRAVENOUS PSA?

	0	1	2	3	4	5	6	7	8	>8
Physician coverage	◯	◯	◯	◯	◯	◯	◯	◯	◯	◯
Nurse coverage	◯	◯	◯	◯	◯	◯	◯	◯	◯	◯

Do you have sets of guidelines/protocols in place for the following ?

	Yes	No	Unsure
TRAINING: Specific PSA curriculum/courses, aside from advanced life support courses, required for the staff administering the sedation	◯	◯	◯
TRAINING: Specific number of supervised PSA before performing PSA solo	◯	◯	◯
SAFETY: General safety rules for administering sedation (which patients can or cannot be sedated in the ED, number of staff members that needs to be present in the room, which monitoring needs to be applied, etc.)	◯	◯	◯
CHECKLIST: A specific checklist, used just prior to the PSA, of material, adjunct medications or information that need to be prepared or obtained, in preparation for the sedation	◯	◯	◯

Do you have non-invasive capnography (through nasal/oral monitoring cannulae) available in your ED?

◯ No
◯ Yes: it is ALWAYS used during intravenous PSA
◯ Yes: it is OFTEN used during intravenous PSA
◯ Yes: it is OCCASIONALLY used during intravenous PSA

Of the following ''problem situations'', how much does each of them apply to your department?

	Never a problem	Sometimes a problem	Often a problem	Always a problem
The doctors are often unavailable to perform PSA (short staffed)	○	○	○	○
The nurses are often unavailable to perform PSA (short staffed)	○	○	○	○
The ANESTHESISTS resist the use of PSA by NON-anesthesists	○	○	○	○
The emergency department PHYSICIANS resist the use of PSA in the ED because of ED crowding	○	○	○	○
The emergency department NURSES resist the use of PSA in the ED because of ED crowding	○	○	○	○
Lack of physical space to perform PSA	○	○	○	○

Which molecules are currently restricted to anesthesiologist use, in your emergency department (meaning you are not free to create a protocol and use them in children without their direct supervision or official approval) ?

- ☐ Nitrous oxide
- ☐ Midazolam
- ☐ Ketamine
- ☐ Propofol
- ☐ Dexmedetomidine
- ☐ Fentanyl
- ☐ No molecule is restricted to anesthesiologist-only use

Of the following statements, please state how much you agree with each:

	Completely disagree	Somewhat disagree	Unsure	Somewhat agree	Completely agree
Systematically using midazolam before ketamine PSA has shown to reduce emergence phenomenon	◯	◯	◯	◯	◯
Ketamine often gives nightmares and behavior changes for several days after administration	◯	◯	◯	◯	◯
Propofol administration in the emergency department is unsafe	◯	◯	◯	◯	◯
Ketamine is a useful agent in the emergency department	◯	◯	◯	◯	◯
I feel comfortable performing an intravenous sedation on a non-emergent ASA PS Class III patient in the ED (ASA PS: American Society of Anaesthesiology Physical Status Classification, example: 3 year old boy with history of severe asthma and who is currently wheezing)	◯	◯	◯	◯	◯
Dexmedetomidine causes neuroapoptosis	◯	◯	◯	◯	◯
One dose of fentanyl of 1 mcg/kg is enough to cause moderate sedation	◯	◯	◯	◯	◯
One physician is enough to simultaneously perform the PSA AND the procedure itself when using Propofol	◯	◯	◯	◯	◯
Systematically using glycopyrrolate or atropine before ketamine PSA decreases airway adverse events	◯	◯	◯	◯	◯

Specialty(ies):<br><br>What are the specialty(ies) of the physicians performing PSA for your emergency department pediatric patients (multiple answers possible)?	☐ General practitioner (no particular specialty)<br>☐ General Paediatrics<br>☐ Internal medicine (adults only)<br>☐ General Emergency Medicine (children and adults)<br>☐ Anesthesiology<br>☐ General Surgery<br>☐ Other
What other specialty is that?	__________________________________
If any, what are the SUB specialty(ies) of the physicians performing PSA in your ED (multiple answers possible)?	☐ Paediatric Emergency Medicine<br>☐ Paediatric Critical Care Medicine<br>☐ Paediatric Anesthesiology<br>☐ Paediatric Surgery<br>☐ No paediatric SUBspecialists performing PSA in our ED<br>☐ Other
What other sub specialty is that?	__________________________________
Board certification:<br><br>In your knowledge, what is the proportion of the physicians performing PSA on children in your ED that hold a certification by your national board (or equivalent), for their specialty ?	◯ 100%<br>◯ 75-99%<br>◯ 50-75%<br>◯ 25-50%<br>◯ 0-25%
Pediatric advanced life support course:<br><br>In your knowledge, what is the proportion of the physicians performing PSA in your Emergency Department that hold a current certification in EPALS (European Paediatric Advanced Life Support), PALS (Pediatric Advanced Life support) or other similar advanced life support course for children?	◯ 100%<br>◯ 75-99%<br>◯ 50-75%<br>◯ 25-50%<br>◯ 0-25%
Are physicians in training usually allowed to perform PSA?	◯ No<br>◯ Yes, all are allowed<br>◯ Yes, if in senior years

In your country, are Emergency Medicine and Pediatric Emergency Medicine recognized by a national board certification?

	Yes	No	Not sure
Emergency Medicine	◯	◯	◯
Pediatric Emegency Medicine	◯	◯	◯

Do you have a protocol which allows nurses to give analgesics from ED triage, without asking the doctor for a signed prescription every time?

◯ No
◯ Yes

For which medications is that protocol in place?

☐ Paracetamol
☐ Ibuprofen or similar nonsteroidal antiinflammatory
☐ Tramadol or similar oral opiate
☐ Intranasal fentanyl
☐ Other

Which other medication is that?

____

Are Child Life Therapists (aka Hospital Play specialists. Not clowns, not nurses' aids) available to you, in the Pediatric Emergency Department?

◯ No
◯ Yes: day and night
◯ Yes: during the busiest hours of the day and evening.
◯ Yes: during normal daytime business hours

Is hypnosis available to the children of your Emergency Department?

◯ No
◯ Yes: day and night
◯ Yes: during the busiest hours of the day and evening
◯ Yes: during normal daytime business hours

Is a topical WOUND anesthetist available for suturing of lacerations, in your department?

◯ No, we do not have one available
◯ No, because we do not see/treat lacerations at our hospital (referred or transported elsewhere from site of injury)
◯ Yes: LET/LAT (lidocaine, epinephrine/adrenaline, tetracaine) gel
◯ Yes: one similar to LET

What is that WOUND local anesthetic called?

____

Is skin glue (cyanoacrylate, such as Dermabond, SurgiSeal, etc) available for closing minor wounds, in your ED ?

◯ No, it is not available
◯ No, because we do not see/treat lacerations at our hospital (referred or transported elsewhere from site of injury)
◯ Yes

Is a topical NEEDLE stick anesthetic available for a blood draw or for the placement of an intravenous line, in your department ?

◯ No
◯ Yes

Which topical NEEDLE stick anesthetic is available in your department?

☐ EMLA (eutectic mixture of local anesthetics)
☐ Ethyl Chloride
☐ Other

What is that other NEEDLE stick anesthetic called?	__________________________________
Do your emergency department physicians USUALLY sedate patients OUTSIDE of the emergency department (for gastroenterology or bronchoscopy procedures, MRI, etc.)?	◯ Yes<br>◯ No
Does your hospital have a Medical Sedation Service OUTSIDE of the operating theater that serves children?<br><br>(Defined as a group of physicians on a pre-defined schedule, who sedate patients from different services of the hospital, such as from hospitalization units, from radiology, etc.) ?	◯ Yes<br>◯ No
Which physicians staff that service (multiple answers possible) ?	☐ Anesthesiologists<br>☐ General Paediatricians<br>☐ Paediatric Intensive Care Unit physicians<br>☐ Paediatric Emergency Medicine physicians<br>☐ Other
What type of physician is that?	____
How satisfied are you currently with the management of pain and anxiety of children during procedures, in your department?	◯ Very UNsatisfied<br>◯ Somewhat UNsatisfied<br>◯ Somewhat satisfied<br>◯ Very satisfied
In a few words, please state the MAIN REASON why you are unsatisfied:	____
What are the official names of the department and hospital you work at?	__________________________________
In which city is your hospital located ?	__________________________________
In which country is your hospital located ?	__________________________________
Last question! How many CHILDREN did your emergency department treat, this last ENTIRE year?	__________________________________
